# Value of Arsenic in the Treatment of Pulmonary Phthisis

**Published:** 1868-06

**Authors:** 


### Miscellaneous.

### Value of Arsenic in the Treatment of Pulmonary Phthisis.

In a memoir read before the Academy of Medicine, by Dr. M. Martin, upon the "Value of Arsenic in the Treatment of Pulmonary Phthisis," he arrives at the following conclusions:

1. Arsenical medication has an effect very marked upon phthisis pulmonalis.

2. Its efficacy is more manifest where the progress of the disease is slow and sluggish than in phthisis accompanied by fever.

3. Phthisis, rapid in its march, or acute in manifestation, is not at all modified by arsenic.

4. In a large number of cases, even advanced into the state of hectic fever, the general condition of the subjects may be favorably modified for quite a lengthy period.

5. The modifications of local lesions are only produced very slowly.

6. A number of recoveries should be attributed to arsenical medication, which would have accomplished more if those patients, who fancying themselves well, had not desisted in the medication.

7. In order to be efficacious, the treatment should be continued for a long time.

8. The arsenic should be administered in fractionally small doses.

9. The daily doses should not exceed two centigrammes, (equivalent to two-tenths gr. apothecarie's.) [M. Moutard Martin employs arsenious acid in granules of one millegramme each.—

Commencing with one or two millegrammes he gradually increases the dose by granule, according to the tolerance of the individual.]

10. Arsenic is better borne by those in whom the disease has made but little progress than by those far advanced in consumption.

11. When the doses can be augmented from 15 millegrammes to 2 centigrammes, the tolerance may be, so to speak, unlimited.

12. The arsenical medication is primarily restorative in its action and secondarily modifies the pulmonary lesion. However, certain facts show that arsenic exercises a direct power over the respiratory function, and acts, doubtlessly, upon the pulmonary tissue itself and upon the tubercule.—*Le Mouvement Medicale*, 1868.

